# Supplementary material for: In Vitro Antioxidant and Anti-Propionibacterium acnes Activities of Cold Water, Hot Water, and Methanol Extracts, and Their Respective Ethyl Acetate Fractions, from Sanguisorba officinalis L. Roots
Source: Molecules. 2018 Nov 16;23(11):3001. doi: 10.3390/molecules23113001 (PMC6278274; doi:10.3390/molecules23113001)
Supplement: Supplementary file 1 [file molecules-23-03001-s001.pdf]

Supplementary

Table S1 Effect of various extracts from roots of *S. officinalis* L. on the bacterial growth determined by paper disc diffusion assay.

**Extract/ Fraction**[a]	*E. coli*	*V. parahaemolyticus*	*L. monocytogenes*	*S. aureus*	*P. acnes*
	**Inhibition zone / 5 mg treatment**				
CWE	-[b]	-	-	-	+
HWE	-	-	-	-	+
ME	+	+	+	+	+

[a] CWE: cold water extract; HWE: hot water extract; ME: methanol extract. [b] No inhibition (-) and Inhibition (+). [c] *Escherichia coli, Vibrio parahaemolyticus, Listeria monocytogenes, Staphlyococcus aureus, Propionibacterium acnes.*

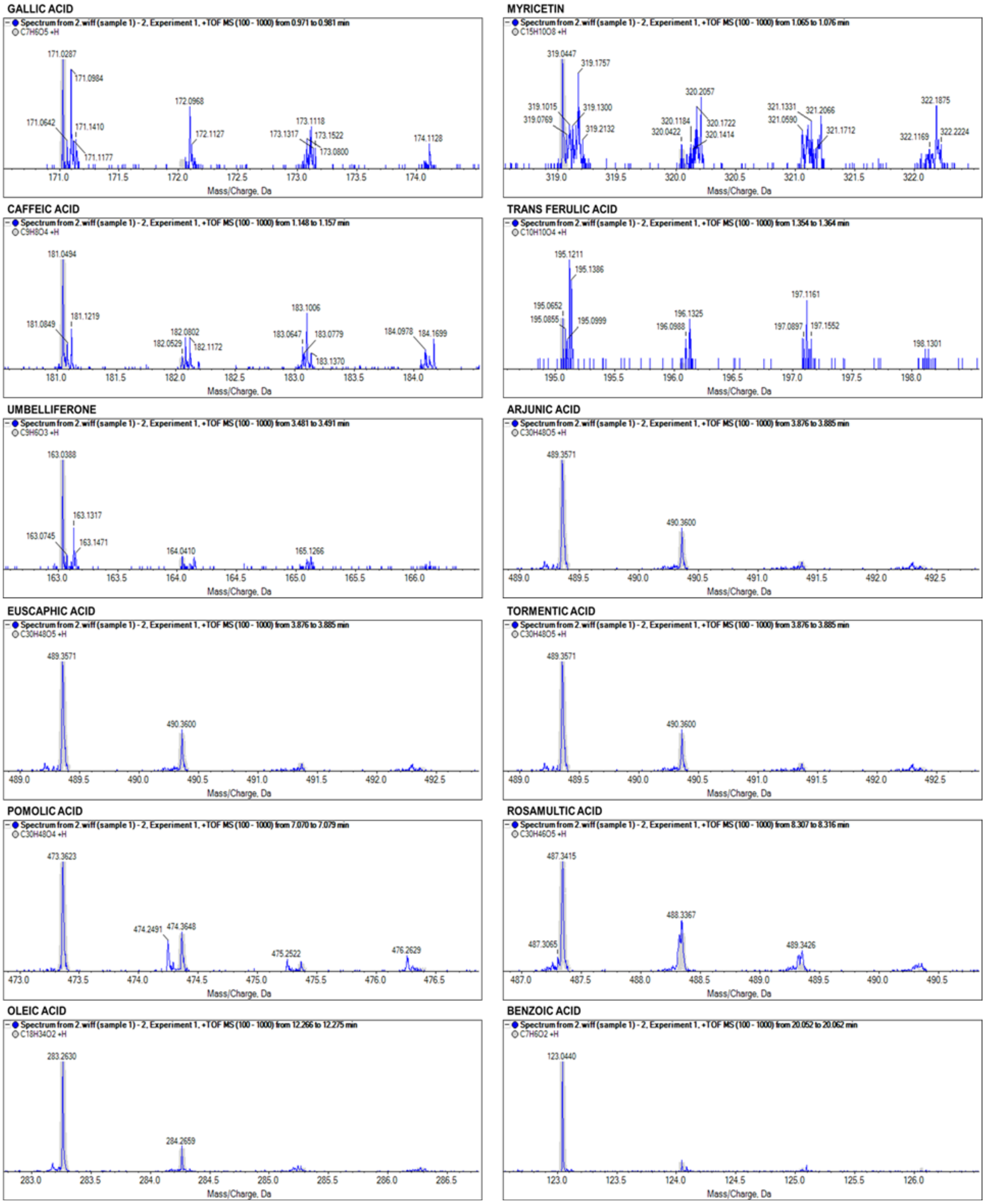

Figure S1 UHPLC-QTOF-MS spectra of major compounds in SOR CWE-EA.

CATECHIN

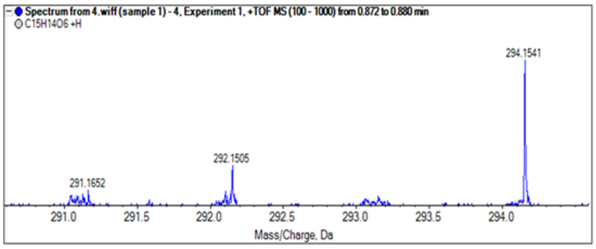

GALLIC ACID

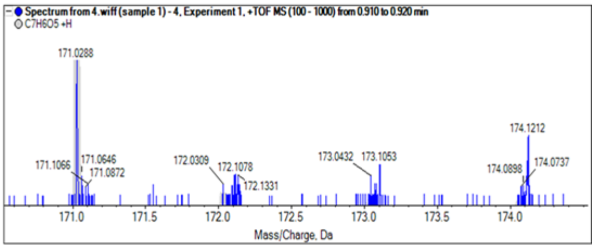

HYPEROSIDE

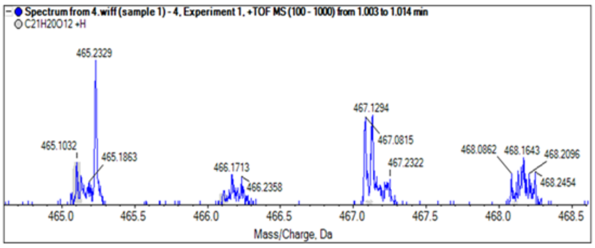

CATECHIN GALLATE

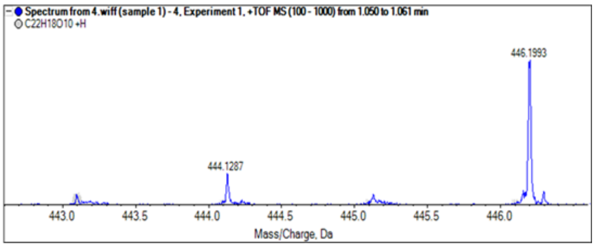

EPICATECHIN GALLATE

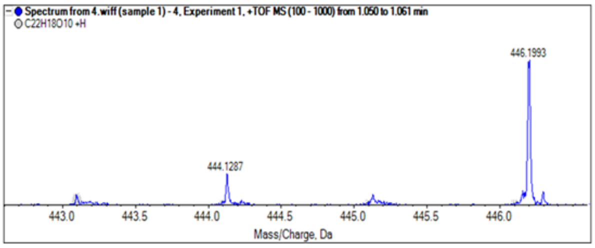

ELLAGIC ACID

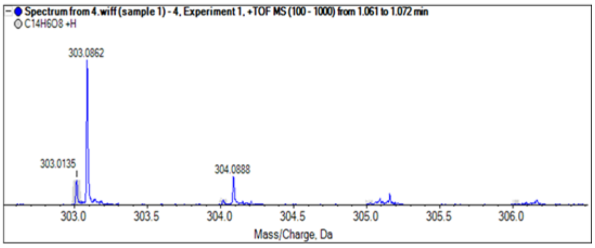

CAFFEIC ACID

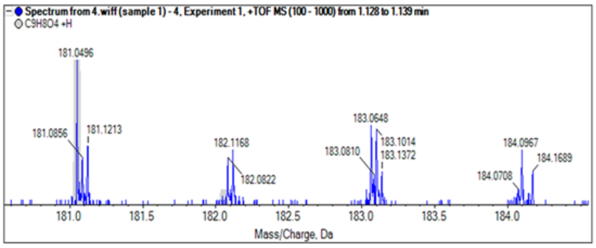

SYRINGIC ACID

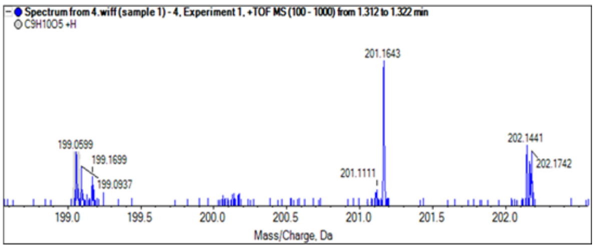

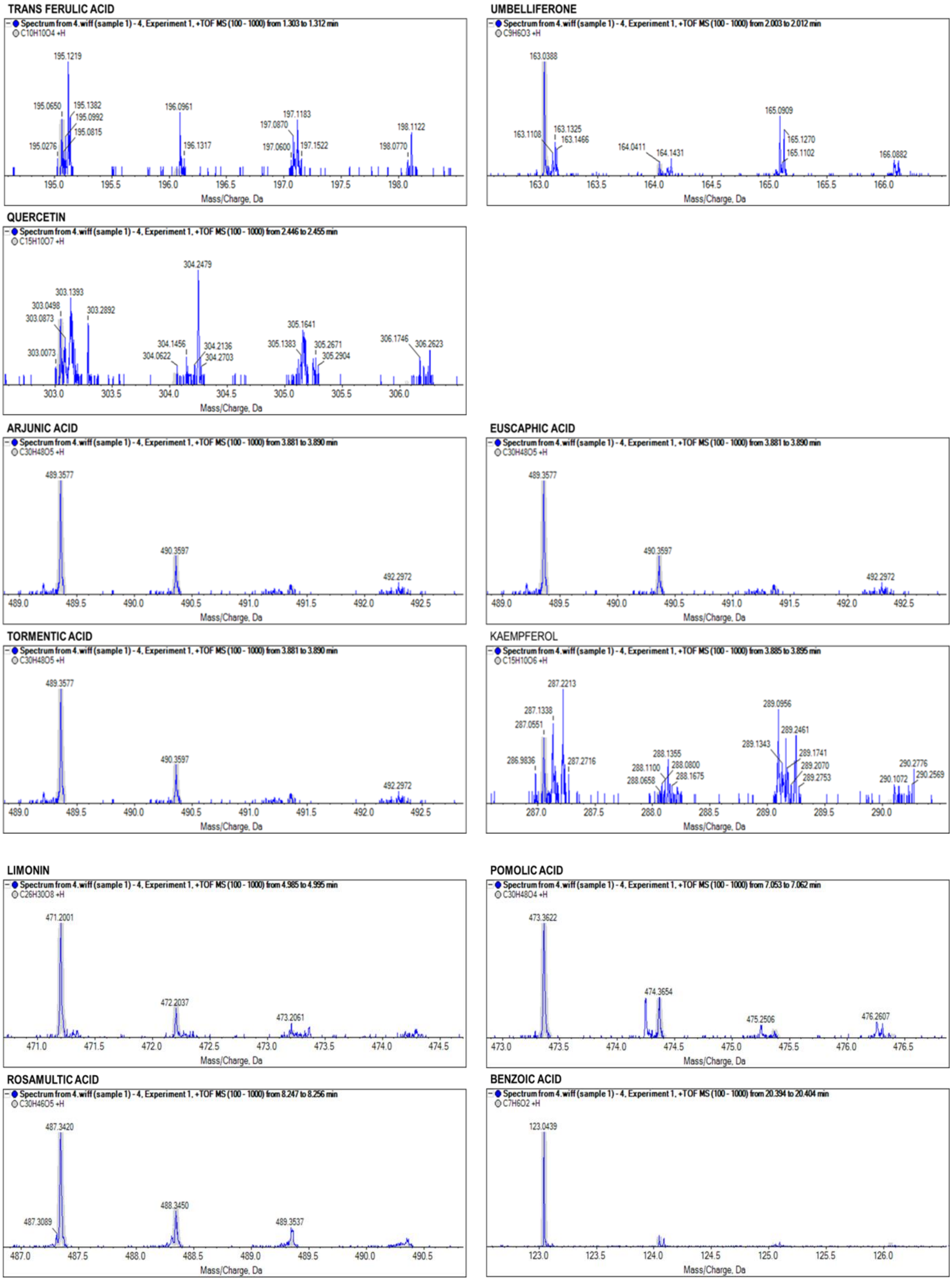

Figure S2 UHPLC-QTOF-MS spectra of major compounds in SOR HWE-EA.
